# Supplementary material for: Development and psychometric properties of the Digital Difficulties Scale (DDS): An instrument to measure who is disadvantaged to fulfill basic needs by experiencing difficulties in using a smartphone or computer
Source: PLoS One. 2020 May 29;15(5):e0233891. doi: 10.1371/journal.pone.0233891 (PMC7259742; doi:10.1371/journal.pone.0233891)
Supplement: S1 Appendix — (DOCX) [file pone.0233891.s001.docx]

**S1 Appendix. Full items of the Digital Difficulties Scale.**

| The Digital Difficulties Scale | M | SD |
| --- | --- | --- |

| Specific Digital Difficulties (SDD) | |  |  |
| --- | --- | --- | --- |
| SDD 1 | If necessary, to what extent would you have difficulty to apply for a subsidy or premium from the government online^a^, without the help of others? (e.g., for parental leave or housing refurbishment) | 1.92 | 1.00 |
| SDD 2 | If necessary, to what extent would you have difficulty to claim benefits from the government online, without the help of others? (e.g., for unemployment, illness, disability) | 1.85 | 0.96 |
| SDD 3 | If necessary, to what extent would you have difficulty to search for job vacancies online, without the help of others? | 1.51 | 0.81 |
| SDD 4 | If necessary, to what extent would you have difficulty to register as unemployed with the government online, without the help of others? | 1.59 | 0.87 |
| SDD 5 | If necessary, to what extent would you have difficulty to apply for jobs online, without the help of others? (e.g., uploading a cv or motivation letter) | 1.57 | 0.88 |
| SDD 6 | If necessary, to what extent would you have difficulty to find information about the services or assistance of social organizations online, without the help of others? (e.g., Public Centre for Welfare) | 1.54 | 0.80 |
| SDD 7 | If necessary, to what extent would you have difficulty to find information about health insurances, fire insurances or family insurances online, without the help of others? (e.g., about the cost or reimbursement) | 1.60 | 0.84 |
| SDD 8 | If necessary, to what extent would you have difficulty to find information about electricity, gas or water online, without the help of others? (e.g., about the cost) | 1.55 | 0.81 |
| SDD 9^b^ | If necessary, to what extent would you have difficulty to find information about healthcare online, without the help of others? (e.g., about a healthy lifestyle or hospital) | 1.53 | 0.77 |
| SDD 10^b^ | If necessary, to what extent would you have difficulty to make payments or transfers online, without the help of others? | 1.35 | 0.71 |
| SDD 11^b^ | If necessary, to what extent would you have difficulty to fill out your tax letter online, without the help of others? | 1.93 | 1.10 |

| General Digital Difficulties (GDD) | |  |  |
| --- | --- | --- | --- |
| GDD 1 | In general, I often have difficulty when using my smartphone, apps, websites, or computer programs | 2.08 | 1.12 |
| GDD 2 | In general, I am not able to solve questions or problems on my own when using my smartphone, apps, websites, or computer programs | 2.33 | 1.19 |
| GDD 3 | In general, I need support when trying out something new on my smartphone or computer | 2.45 | 1.29 |
| GDD 4 | In general, I find it hard to adjust settings of my smartphone, apps, websites, or computer programs (for example, privacy or safety settings) | 2.34 | 1.26 |
| GDD 5 | In general, I often have questions or problems when using my smartphone, apps, websites or computer programs after an update has been done | 2.32 | 1.19 |
| Worries about Future Digital Difficulties (WFDD) | |  |  |
| WFDD 1 | In the past six months, how often did you worry that you will be unable to keep up with ongoing changes in smartphones, apps, websites or computer programs in the future? | 2.26 | 1.17 |
| WFDD 2 | In the past six months, how often did you worry that future developed smartphones, apps, websites or computer programs will be too difficult for you to use? | 2.28 | 1.16 |
| WFDD 3 | In the past six months, how often did you worry that you will find it hard to keep up with using smartphones, apps, websites or computer programs in the future? | 2.33 | 1.20 |

Mean and SD of the items are based on sample 3 (n = 1000).

^a^online = using an app or website.

^b^item is excluded from the final instrument based on exploratory factor analysis.

Answers for SDD items were 1 = Having no difficulty, 2 = Having rather no difficulty, 3 = Having rather difficulty, 4 = Having difficulty.

Answers for GDD items were 1 = Disagree, 2 = Rather disagree, 3 = Neither disagree, neither agree, 4 = Rather agree, 5 = Agree.

Answers for WFDD items were 1 = (Almost) never, 2 = Rarely, 3 = Sometimes, 4 = Often, 5 = Very often.
